# Multiscale reconstruction of bronchus and cancer cells in human lung adenocarcinoma

**DOI:** 10.1186/s12938-023-01072-4

**Published:** 2023-02-08

**Authors:** Xin Li, Bowen Zhang, Yanmei Liang, Ting Li

**Affiliations:** 1 Department of Thoracic Surgery, Tianjin Chest Hospital (Affiliated Hospital of Tianjin University), Tianjin, China; 2 Institute of Biomedical Engineering, Chinese Academy of Medical Sciences and Peking Union Medical College, No.236 Baidi Road, Nankai District, Tianjin, 300192 China; 3 Institute of Modern Optics, Tianjin Key Laboratory of Micro-Scale Optical Information Science and Technology, Nankai University, Tianjin, China

**Keywords:** Lung adenocarcinoma, Three-dimensional, fMOST, Propidium iodide

## Abstract

**Background:**

While previous studies primarily focused on the structure of the normal whole mouse lung, the whole bronchus and cytoarchitectural details of the mouse intact lung lobe have been discovered at single-cell resolution. Revealing the sophisticated lung adenocarcinoma structure at three-dimensional (3D) and single-cell level remains a fundamental and critical challenge for the pathological mechanism research of lung adenocarcinoma (LA).

**Methods:**

Fluorescence micro-optical Sectioning Tomography (fMOST) combined with PI staining were used to obtain the 3D imaging of the human LA tissue at single-cell resolution.

**Results:**

With a spatial resolution of 0.32 × 0.32 × 1.0 μm^3^, the dataset of human LA with single-cell precision consists of two channels, each of which contains information about the bronchi and the cytoarchitecture. The bronchial wall is thicker and the lumen is smaller in the cancer tissue, in which its original normal structure is vanished. More solid components, more clustered cancer cells with larger nucleoli, and more significant atypia are found in cancer tissue. In paracancerous tissue, the bronchial wall cells have a monolayer or bilayer structure, cluster along the wall, and are relatively dispersed. Few fibrous structures and occasional dissemination of spread through air spaces (STAS) are observed.

**Conclusions:**

Based on the human LA tissue dataset obtained by fMOST and PI staining, the bronchi and cells were reconstructed and visualized. This work provides a technical roadmap for studying the bronchus and cytoarchitectural structure and their spatial relationship in LA tissue, which may help with the understanding of the main histological structure of LA among pathologists.

**Supplementary Information:**

The online version contains supplementary material available at 10.1186/s12938-023-01072-4.

## Introduction

Lung cancer is one of the serious public health problems worldwide [1]. About 85% of all cases of lung cancer are non-small cell lung cancer (NSCLC). Due to the absence of specific symptoms in patients with early NSCLC, approximately 70% of NSCLC patients were already in advanced stage once diagnosis, accompanied by local or distant metastases (stage III and IV), which led to a 5-year overall survival (OS) rate of only 10–15% [2]. However, the 5-year OS rate of NSCLC patients at early-stage after surgery is 60–90%. In addition, patients with NSCLC also have a high likelihood of disease impoverishing and the incidence of catastrophic health expenditures is as high as 82.3% [3]. Lung adenocarcinoma (LA) accounts for 68.6% of NSCLC. In recent 20 years, the incidence of LA in China is the highest among people under the age of 49, posing an immeasurable loss of human resources to the society [4].

According to the 2015 World Health Organization (WHO) classification of lung adenocarcinoma, lung adenocarcinoma is divided into five subtypes: micropapillary, lepidic, acinar, solid and papillary. Classification and stratification according to the primary structure of lung adenocarcinoma can predict the efficacy of adjuvant chemotherapy [5]. These five subtypes are classified into three prognostic groups based on the pathology: low grade (mostly lepidic), intermediate grade (primarily acinar or papillary) and high grade (primarily solid or micropapillary) [6]. LA is histologically heterogeneous, displaying an aggregate of multiple structures and proportions.

The acinar type is the most prevalent (40–50%) and has the greatest prognostic spectrum when categorized solely by main structures [7]. However, some pathologists may classify these structures as high grade (solid) or intermediate grade (acinar) due to a lack of knowledge about these histological features, leading to uncertainty in tumor classification. Currently, the reproducibility of histological structure assessment of lung adenocarcinoma by multiple pathologists is a challenge [8]. In the study by Moreira AL et al. [9], the average Kappa value was 0.84 + 0.04 and the range of the Kappa value evaluating the grading agreement between the two observation groups (a total of 10 pathologists, 23 cases) was 0.79–0.89. Most of the inconsistent attributed to the distinction between the lepidic and papillary structures, as well as variations in the proportion of high-grade structures. Therefore, the understanding of the main histological structure of lung adenocarcinoma can improve the repeatability of lung adenocarcinoma grading and classification among pathologists, which may ultimately lead to more accurate patient diagnosis, treatment, and prognosis guidance in the future [9].

Currently, pathological section is still the gold standard for clinical diagnosis of LA. Considering the limitations of traditional two-dimensional (2D) images, Memorial Sloan Kettering Cancer Center [10] adopted Whole Slide Imaging (WSI) technology for three-dimensional (3D) reconstruction of LA. However, revealing the sophisticated structure of LA at 3D, and single-cell level remains a fundamental and critical challenge for the pathological mechanism research of lung adenocarcinoma. Here, taking the advantage of the Fluorescence micro-optical Sectioning Tomography (fMOST) combined with PI staining [11], the high-precision, cross-scale visualization of human LA tissue at single-cell resolution was obtained, What's more, the sophisticated architectures and the region-specific distribution patterns in human LA were discovered simultaneously for the first time.

## Results

### Chest-enhanced CT image and postoperative pathology of tumor

**Fig. 1:**
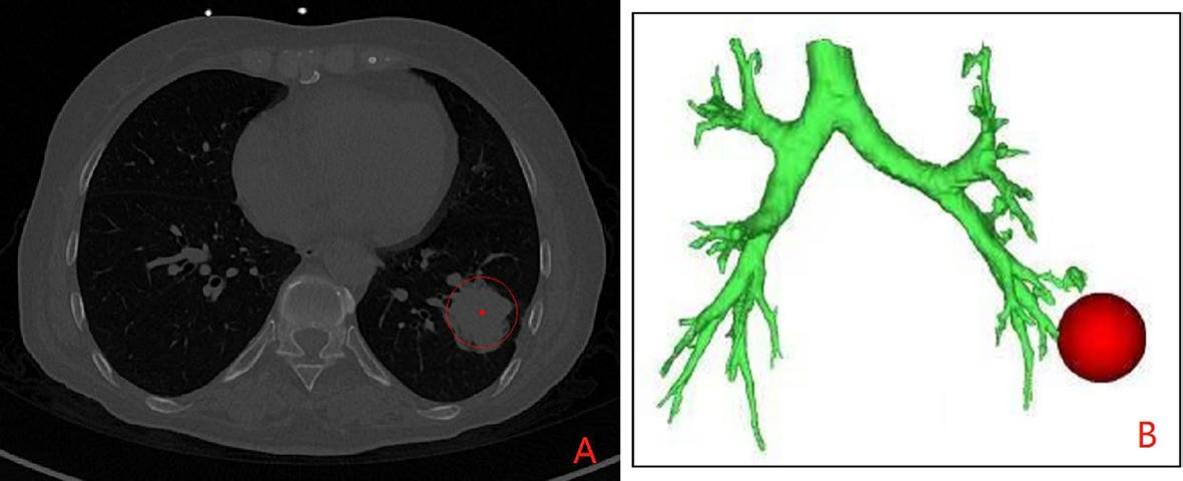
Preoperative location of the tumor (**A** is the max image of tumor on chest-enhanced CT, **B** is the three-dimensional reconstruction of bronchi and tumor based on chest-enhanced CT)

Mimics Medical 20.0 software was used for three-dimensional reconstruction of bronchi and tumor based on chest-enhanced CT. The results showed that the tumor was located in the basal segment of the lower lobe in the left lung, the tumor was 4.2 cm × 3.5 cm × 3 cm in size, and peripheral lung cancer was considered first (Fig. 1).

**Fig. 2:**
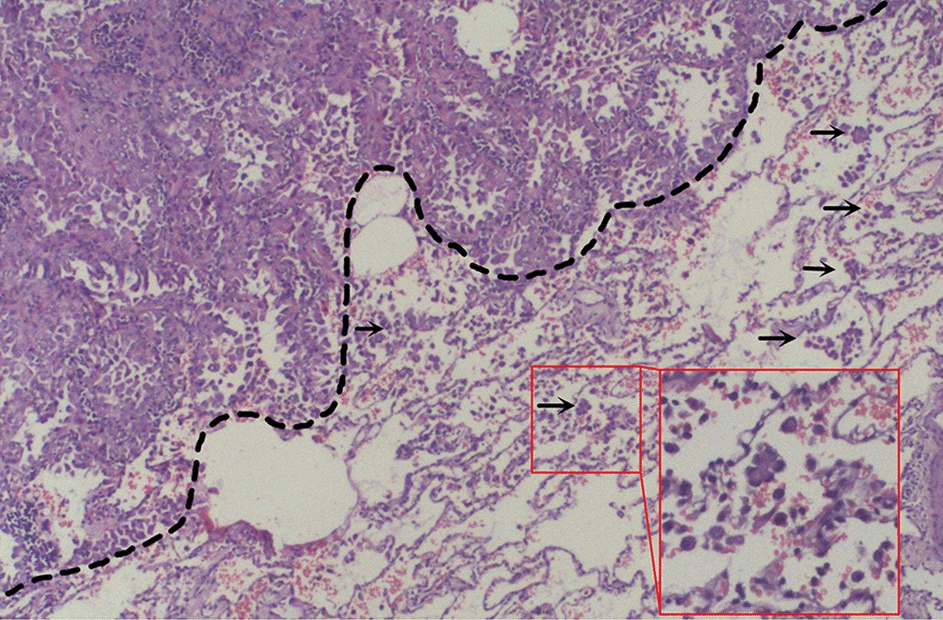
Hematoxylin–eosin (HE) staining for pathological section (the black arrows indicated the STAS and STAS was observed in 200 × light microscope)

The patient was followed up for 7 months after surgery with no obvious abnormalities and no specific symptoms. Postoperative pathology was shown as follows: adenocarcinoma, lepidic type accounts for 60%, acinoid type accounts for 20%, micropapillary type accounts for 20%, 4.2*3.5*3 cm, the mass invaded the visceral pleura. No cancer metastasis was observed in lymph nodes of group 5, 7, 9, 10 and 11, while metastasis was observed in lymph nodes of group 12 and 13 (1/1, 1/1), T2aN1M0, stage IIB. HE stain was observed under 20 × light microscope, adenocarcinoma cell nests and fibrotic stroma could be observed, as shown in Fig. 2. The boundary between the cancer and paracancerous tissues was outlined by a black dotted line. Multiple STAS and prominent bronchoalveoli were visible in the paracancerous tissue. In addition, more alveoli and more dispersed cells were observed in paracancer tissue.

### Dataset of human LA tissue with single cell resolution

**Fig. 3:**
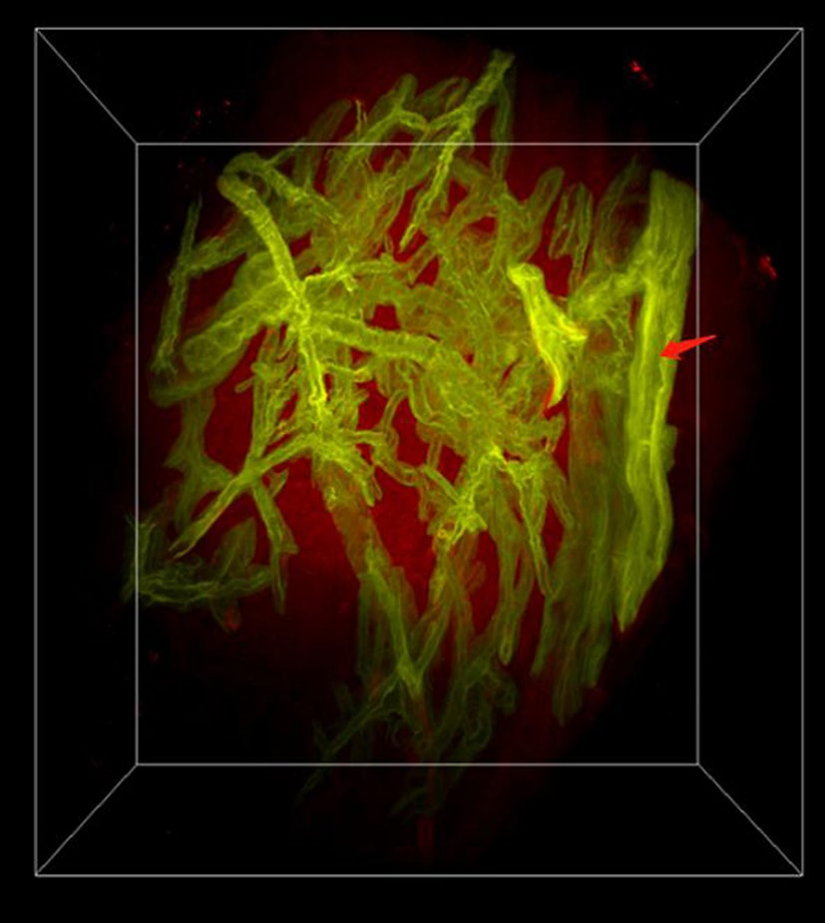
Dual-channel merged image of lung adenocarcinoma tissue, the red arrow is part of longitudinal section of one bronchus, the other levels of bronchus are transverse sections

The fMOST system combined with PI-staining were utilized to obtain dataset of LA tissue. The acquired dataset consists of two channels containing information about the bronchi and the cytoarchitecture, with the spatial resolution of 0.32 × 0.32 × 1.0 μm^3^. In the bronchi channel (green, Additional file 1: Figure S1), we could observe the 3D structure of bronchi and implement the colocalization of the bronchi and cytoarchitecture. In the cytoarchitecture channel (red, Additional file 2: Figure S2), the morphologies of nucleus of all cells can be observed. We merged the two channel images of the same local area to obtain a 3D bird's-eye view containing 4000 sections to visualize and segment the complete structure within this adenocarcinoma tissue, including all airways and cell nests. The specific structures of bronchi, respiratory bronchioles, terminal bronchioles, alveoli, and alveolar sacs were distinguished (Additional file 3: Movie S1). In the Fig. 3, bronchioles with irregular morphology and structure were distributed in the lung adenocarcinoma cancer tissue, the terminal branches of the bronchus, bronchiole, and terminal bronchiole end in the alveoli. Spongiform structures formed by millions of air-filled alveoli were seen in the paracancerous tissue.

### Morphological features of bronchus in cancer and paracancerous tissues

**Fig. 4:**
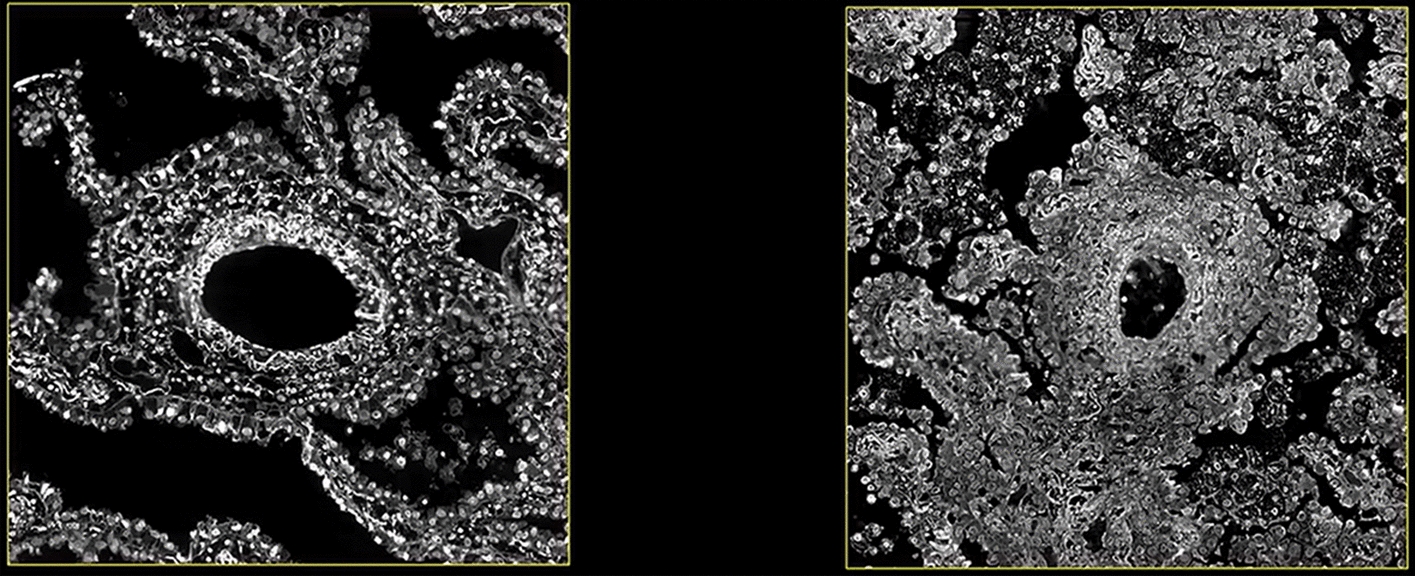
The structure of a bronchus in paracancerous tissue (left) and cancer tissue (right)

A bronchus was tracked and located, and the anatomy of the bronchial wall was observed in cancer and paracancerous tissues (Additional file 4: Movie S2). As shown in Fig. 4 (right), the bronchial wall was thicker and the lumen was narrower in the adenocarcinoma tissue, in which its original normal structure had vanished. The cancer cells adhered to the wall, gathered and proliferated. There were many cells in the lumen, which may be macrophages or exfoliated cancer cells. In the paracancerous tissue, original normal structure of the bronchus was still retained, and the cells were monolayer or bilayer structure, that grew around the wall and were moderately scattered. More vesicle-like tissue were observed around the bronchus.

### Distribution of the cells in cancer and paracancerous tissues

**Fig. 5:**
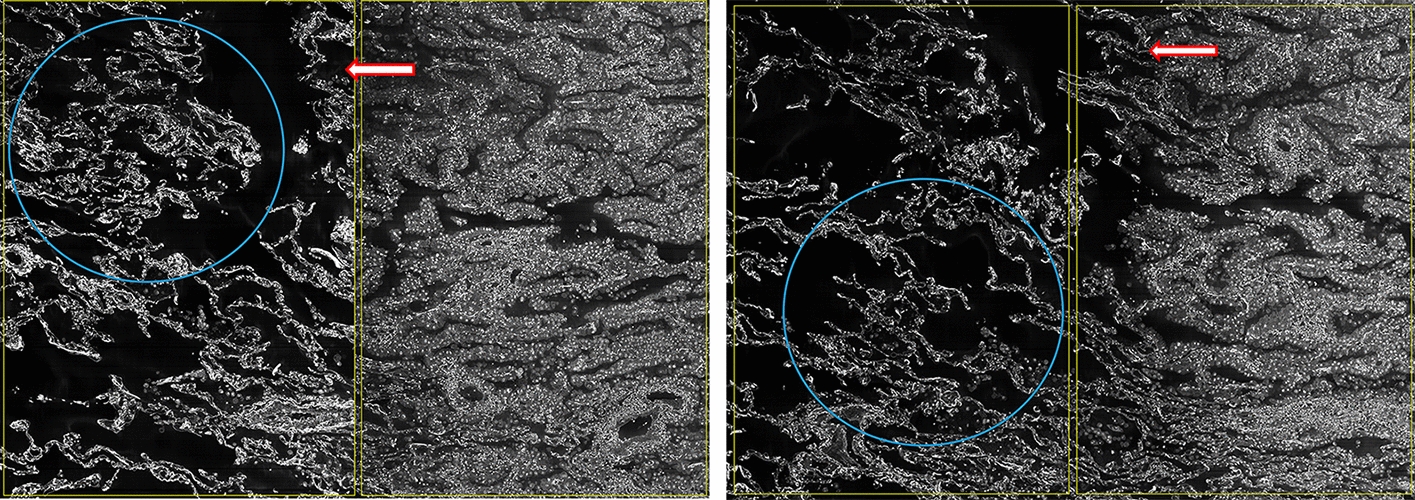
Cellular structure of cancerous and paracancerous tissues. The blue circles are spongiform network, the red arrows are STAS

Compared with paracancerous tissue, more solid components, more clustered cancer cells with larger nucleoli and more significant atypia were discovered in cancer tissues. In the cancer tissues, the cancer cells were cuboidal or columnar, attached to the papillary structure containing the fiber axis, and convex into the alveolar cavity. During the transition from cancer tissue to paracancerous tissue, less solid components, more alveolar-like structures, and thinner lumen walls were observed. In the paracancerous tissue, the cancer cells grew along the structure of the original alveolar wall, mostly monolayer, few fibrous structures and STAS were observed. STAS were found in the alveolar parenchyma extend in a continuous manner within air spaces of the image beyond the edge of the tumor. Alveolar epithelial cells in the paracancerous tissue formed acinar nets, which were scattered and had large voids, becoming alveoli. These alveolis were sparsely located around the respiratory bronchioles and were interconnected to form a large spongiform network (Fig. 5). The alveolar walls were smoother than the airways. The alveoli in the entire lobe were connected to each other through small pores.

## Discussion

3D reconstruction has been extensively used in the medical field due to the quick development of imaging and 3D technologies [12–14]. 3D reconstruction can transform 2D images of the pulmonary bronchi into 3D images of the bronchial tree [15–17], thus the structure of the bronchi and the anatomical variations of the bronchus can be clearly observed. Due to the existence of various differentiation degrees in lung adenocarcinoma tissue, it is impossible to obtain the entire structure and process of pathological development of lung adenocarcinoma by conventional pathological sections. For the first time, a complete 3D reconstruction of lung adenocarcinoma at single-cell resolution was achieved in this study using fMOST combined with PI staining. The Semi-automatic video based on fMOST slice data can quickly and precisely reflect the branches, forms, changes in cell morphology, and number of the lung bronchi, breaking through the limitation that traditional 3D visualization software can only form static simulation. We observed actually distribution of bronchi, respiratory bronchioles, terminal bronchioles, alveoli, and alveolar sacs in accordance with the morphology disparities among various bronchus. Therefore, 3D reconstruction of lung adenocarcinoma tissue at the single-cell resolution based on fMOST can be more tridimensional, more real, clearer and more intuitive to show the detailed information and location of the lesion [18, 19].

In the cancer tissue, the bronchial wall was thicker and the lumen was narrower, and its original normal structure was disappeared. More solid components, more clustered cancer cells with larger nucleoli and more significant atypia were found in cancer tissue. Tumors with solid component appear to behave more aggressively, manifest with a higher stage at presentation [10, 20–22]. In addition, the cancer cells were cuboidal or columnar, attached to the papillary structure containing the fiber axis, and convex into the alveolar cavity. During the transition from cancer tissue to paracancerous tissue, less solid components, more alveolar-like structures, thinner lumen walls, and dissemination of STAS were observed. Glandular structures (high-grade acini) were associated with tumor necrosis and lymphovascular invasion [23]. Moreover, there is proof that patients who have these intricate glandular structures and are STAS positive have a higher recurrence rate and worse survival [24]. The patient was followed up for 7 months after surgery. At present, the patient has no obvious abnormalities and no specific symptoms after routine outpatient reexamination. According to the findings of this investigation, we will continuously monitor this patient to track her prognosis.

There are several challenges in addressing complete 3D imaging of human lung adenocarcinoma tissue. Due to the morphology, diameter and length of lung bronchus varied greatly from cancer tissue to paracancerous tissue, the first challenge is how to scan the multiscale bronchus of the adenocarcinoma tissue with single-cell resolution. Currently, the highest resolution imaging technique of the lung tissue is WSI [10], Whilst, since the WSI protocol involved tissue sectioning, immunostaining, imaging, and finally image stitching, the tomographic sections existed some deformation inevitably during the process, which has a certain impact on the subsequent image registration and 3D reconstruction. While fMOST technology can perfectly solve this problem, this staining-then-sectioning method is not only more convenient and time-saving than WSI, the 3D imaging based on the staining-then-sectioning method can effectively identify the pathological structure of lung cancer.

The second problem is how to complete the staining of a 1cm^3^ human lung cancer tissue. Propidium iodide is a typical fluorescent nucleic acid dye that is frequently used to label DNA and RNA and distinguish between normal and tumor cells by analyzing the cell karyotypes, making it the perfect way to illuminate all of the cells in a tissue or even a large organ during fluorescence imaging. In addition, PI staining adopts the technique of imaging while sectioning, avoiding the problem of imaging inhomogeneity, which is the most typical problem in the process of tissue staining. However, PI staining shows no difference in all nuclei, so it is impossible to distinguish and track various cells in cancer tissue and paracancerous tissue according to the results. Therefore, further research into the proper staining technique for human lung adenocarcinoma tissues is required. HE staining is currently being used by the research team working on this endeavor, with some early results.

There is a limitation with this study. Due to the high cost of tissue preparation and fMOST imaging, it is not available to conduct imaging analysis with multiple samples at the same time. Therefore, a typical human stage II lung adenocarcinoma was carefully selected, and 4000 continuous section images were analyzed with the fMOST system to obtain a continuous non-destructive imaging. This is the first study on the tissue structure of human lung adenocarcinoma, overcoming the bottleneck problem of incomplete imaging due to the need for splicing of traditional continuous imaging. fMOST is the most fine-grained three-dimensional imaging technology at the mesoscopic scale, which has been extensively used in the fields of neural mechanism research, brain and cardiovascular disease research, pathologic toxicology and so on. For example, Qi Zhang completed the multi-scale reconstruction of various blood vessels in the hepatic lobules of a mouse’ liver using fMOST imaging [25]. Several papers have successfully obtained the whole brain imaging of a single mouse brain at subcellular resolution based on fMOST imaging. Those studies demonstrated the ability of fMOST to quantitatively acquire pyramidal dendritic spines and axonal buckles associated with synaptic connections on a brain-wide scale at the whole single-neuron level, as well as mouse whole-brain imaging at the subcellular resolution [26, 27], establishing the foundation for further investigation into the neuronal circuitry underlying the crucial behavioral functions of VP [28]. Therefore, imaging based on a single human lung adenocarcinoma tissue is sufficient to explore the fine structure and pathological progress of lung adenocarcinoma.

## Conclusion

The fMOST system with PI staining was successful in rendering the lung structure in this investigation, and multi-scale co-reconstruction of lung adenocarcinoma tissue was achieved. The combination of fMOST imaging and radiomics technology can improve the surgeon’s overall understanding of the target region [29], and further promote the cognition of lung cancer histological structure and even the development of thoracic surgery.

## Material and methods

### Study case

A 65-year-old female was referred to Tianjin Chest Hospital on Feb 7, 2022 due to flustered and weak for more than 1 month. Chest-enhanced CT showed a mass occupied the lower lobe of the left lung and lung cancer was primarily considered. This patient underwent lobectomy by VATS, the mass was completely removed. The postoperative pathological outcome was adenocarcinoma and the mass invaded the visceral pleura. The patient was followed up regularly after surgery.

### Tissue preparation

A 1 × 1 × 1 cm^3^ specimen was collected within 30 min after the removal of the surgical mass. The specimen was taken from the center of tumor lesions as far as possible, and the regions of liquefaction, necrosis, purulent discharges, and mucus were avoided. Typical LA tissue was sarcomatoid, white or gray in color. A small piece of tissue was scraped from the surface of a 1 × 1×1 cm^3^ tissue sample, which was then placed into a freeze-storage tube filled with 4% paraformaldehyde, and the tube was placed in a freezer at 4 °C for post-fixation. The scraped fragment was stained with HE for pathological analysis. Two professionals and technicians with more than 20 years of experiences examined the sections back-to-back and confirmed that the pathology was typical lung adenocarcinoma, with the effective area of cells in the visual field accounting for more than 80% as the qualified sample. After post-fixation and dehydration, the tissue was immersed in a Lowicryl HM20 series (Ted Pella Inc., Redding, CA, USA), containing 0.2% SBB (70%, 85%, and 100% HM20 for 2 h each and 100% HM20 overnight). The sample was then impregnated in a prepolymerization HM20 solution for 3 days at 4 ℃ and embedded in a vacuum oven at 50 ℃, 24 h.

### LA tissue imaging

LA tissue imaging was performed on f-MOST system (Institute of Biomedical Engineering, Chinese Academy of Medical Sciences and Peking Union Medical College). The prepared sample was immobilized anterior–posterior in a water bath on a 3D translation stage prior to imaging. The sample was submerged in a solution of propidium iodide (PI) and 0.01 M Na_2_CO_3_ to provide a matching refractive index for the objective lens during imaging.

Throughout data collection, the liquid level of the solution was kept above the bottom surface of the objective lens. For wide-field high volume tomography, sectioning was accomplished utilizing a fixed diamond knife and a 3D translation stage. The imaging plane was commonly positioned at 1 µm below the surface to prevent the compromising effects of sectioning markings on the machined surface. Prior to data collection, we concentrated on the sample's top surface and made necessary adjustments to ensure a clear image. We then lowered the sample to position the imaging plane below the specimen's surface. The WVT system automatically carried out the sectioning and imaging after the imaging settings were established to finish the tissue-wide data collection. Additionally, we made flexible adjustments to the image parameters, such as the area of interest and exposure time. To maintain a flattened section, get rid of the cutting chips, clean the PI solution, and keep a consistent PI concentration, we also used a recirculating filtering equipment.

The objective performed a line-scanning block-face scan of the sample's surface at the depth of 1 µm. To cover the entire coronal plane in each layer imaging, we used a strip-scanning (x axis) model along with a montage in the y axis. The fluorescence was captured with a TDI-CCD camera after being gathered using a microscope objective, passing through a bandpass filter. The diamond knife removed the photographed surface from one surface when it had been finished, exposing the smooth, brand-new surface for imaging. To get the required dataset, we repeated these steps across the entire sample volume.

Last but not least, the fMOST system carried out a number of cycles of automatic sectioning with an axial step size of 1 µm, then imaging at a voxel size of 0.32 × 0.32 × 1.0 µm^3^ with one channel for PI-stained cytoarchitecture and another channel for Alexa fluor 488, which were simultaneously detected using two cameras. The raw data acquisition lasted continuously for more than 2 weeks and the raw dataset was larger than 8 terabytes, including 4000 coronal sections for each channel.

The BioMapping7500 system (Institute of Biomedical Engineering, Chinese Academy of Medical Sciences, and Peking Union Medical College), which consists of a 20X Olympus microscope objective, one laser with a wavelength of 561 nm, and a TDI-CCD camera, was used to detect the images from fMOST.

### Processing of images

#### Preprocessing of images

Two detection channels performed all image preprocessing of the obtained data. Based on precise spatial orientation and nearby overlap, the stripes of each coronal portion were stitched to generate a full section. Section by section, lateral illumination adjustment was carried out. Calculating the mean intensity along each direction and fitting the relevant polynomial curves led to the determination of the correction coefficient along each direction. By calculating the images’ average grey-scale values, it was possible to adjust for axial lighting by balancing the brightness of the various coronal portions. Finally, using LZW compression, we created a single image at the imaging plane for image storage in a 16-bit depth TIFF format. Matlab and C +  + were implemented to preprocess obtained images. On a computer server with 32 cores operating at 2.9 GHz per core, image preprocesses for the LA tissue data set at the voxel resolution of 0.32 × 0.32 × 1.0 µm^3^ were carried out.

#### Reconstruction and visualization

To create the charts and movies, we used the Amira software (version 2020.1, FEI, Merignac Cedex, France) and the Imaris software (version 9.7.2, bitplane, Switzerland) to visualize the dataset. Amira software was used to import the preprocessed dataset using a desktop graphical workstation (7920 with one Intel Xeon Gold 6226R CPU, 512 GB memory and an Nvidia GeForce RTX 3090 graphics card, Dell Inc., Round Rock, Texas, USA). We used the OTSU thresholding technique in conjunction with manually corrected parameters on the red channel cytoarchitecture pictures to reconstruct the bronchus. Amira was used to convert the TIFF data format to the native LDA type so that the TB-sized data could be processed on a single workstation. The extraction of the data in the range of interest, sampling or interpolation, reslicing of the images, determination of the maximum intensity projection, volume and surface rendering, and creation of movies using Amira's main module were all steps in the visualization process [30, 31].

### Quality control

The quality of samples directly determines the success of fMOST imaging, so the sample should be selected strictly. A small piece of tissue was scraped off the surface of the tissue and made into HE sections for pathological examination. The sections were examined back-to-back by two professional and technical personnel of the department of pathology, and the typical stage II lung adenocarcinoma were confirmed as qualified samples.

## Supplementary Information

**Additional file 1.** Imaging of bronchi channel of human lung adenocarcinoma tissue.

**Additional file 2.** Imaging of cytoarchitecture channel of human lung adenocarcinoma tissue.

**Additional file 3.** 3D bird’s-eye view of human lung adenocarcinoma tissue.

**Additional file 4.** A bronchus was tracked and located in paracancerous (left) and cancer (right) tissue respectively.

## Data Availability

The datasets supporting this study's findings are included in the publication and its supplemental materials. On reasonable request, the corresponding author will provide raw data.

## Abbreviations

3D: Three dimensional
LA: Lung adenocarcinoma
fMOST: Fluorescence micro-optical sectioning tomography
STAS: Spread through air spaces
PI: Propidium iodide
NSCLC: Non-small cell lung cancer
OS: Overall survival
WHO: World Health Organization
2D: Two dimensional
WSI: Whole slide imaging
HE: Hematoxylin–eosin
VP: Ventral pallidum
