# Supplementary figures and images for: Multiscale reconstruction of bronchus and cancer cells in human lung adenocarcinoma
Source: Biomed Eng Online. 2023 Feb 8;22:11. doi: 10.1186/s12938-023-01072-4 (PMC9906908; doi:10.1186/s12938-023-01072-4)

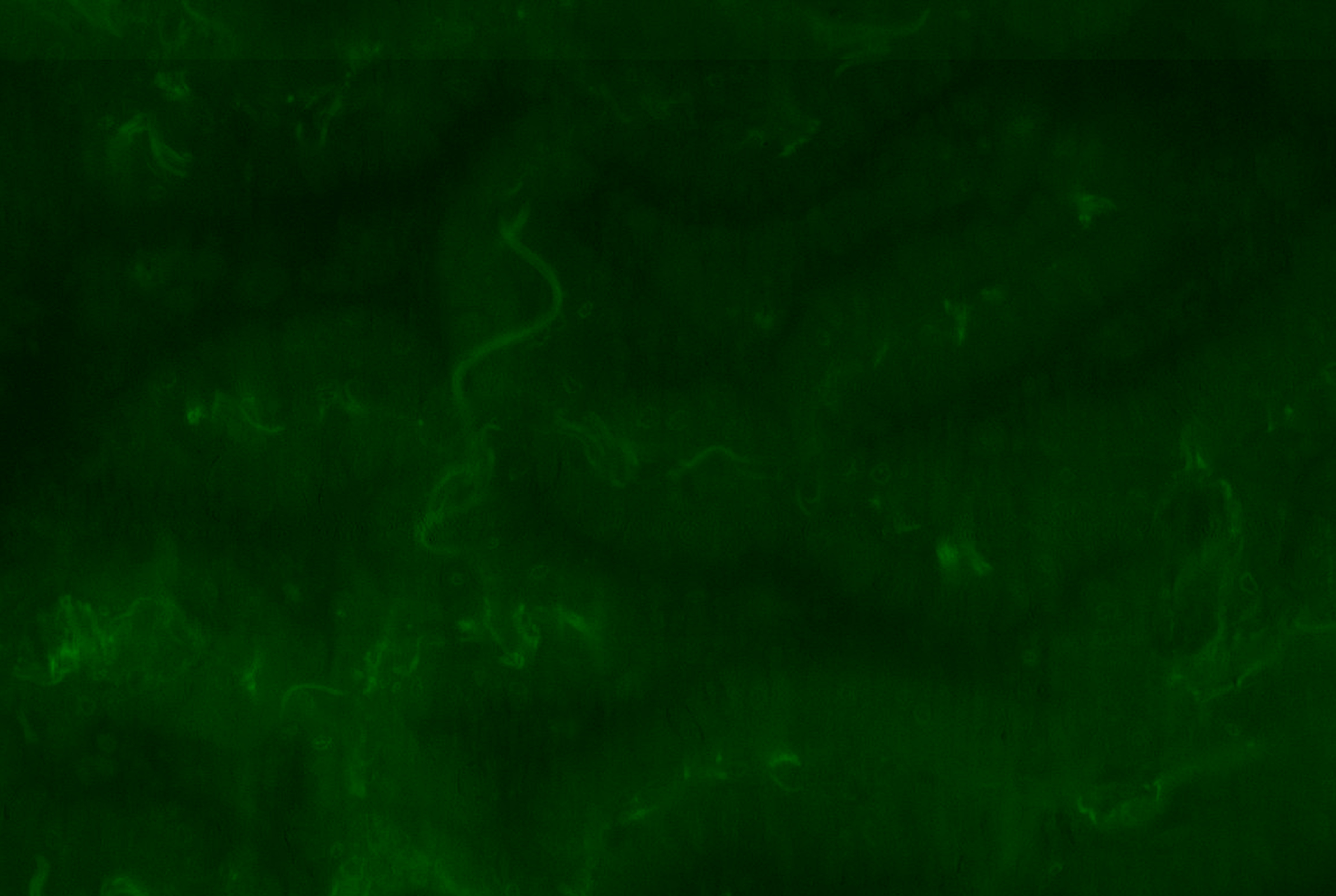

Supplement: Supplementary file 1 — Additional file 1. Imaging of bronchi channel of human lung adenocarcinoma tissue. [file 12938_2023_1072_MOESM1_ESM.jpg]

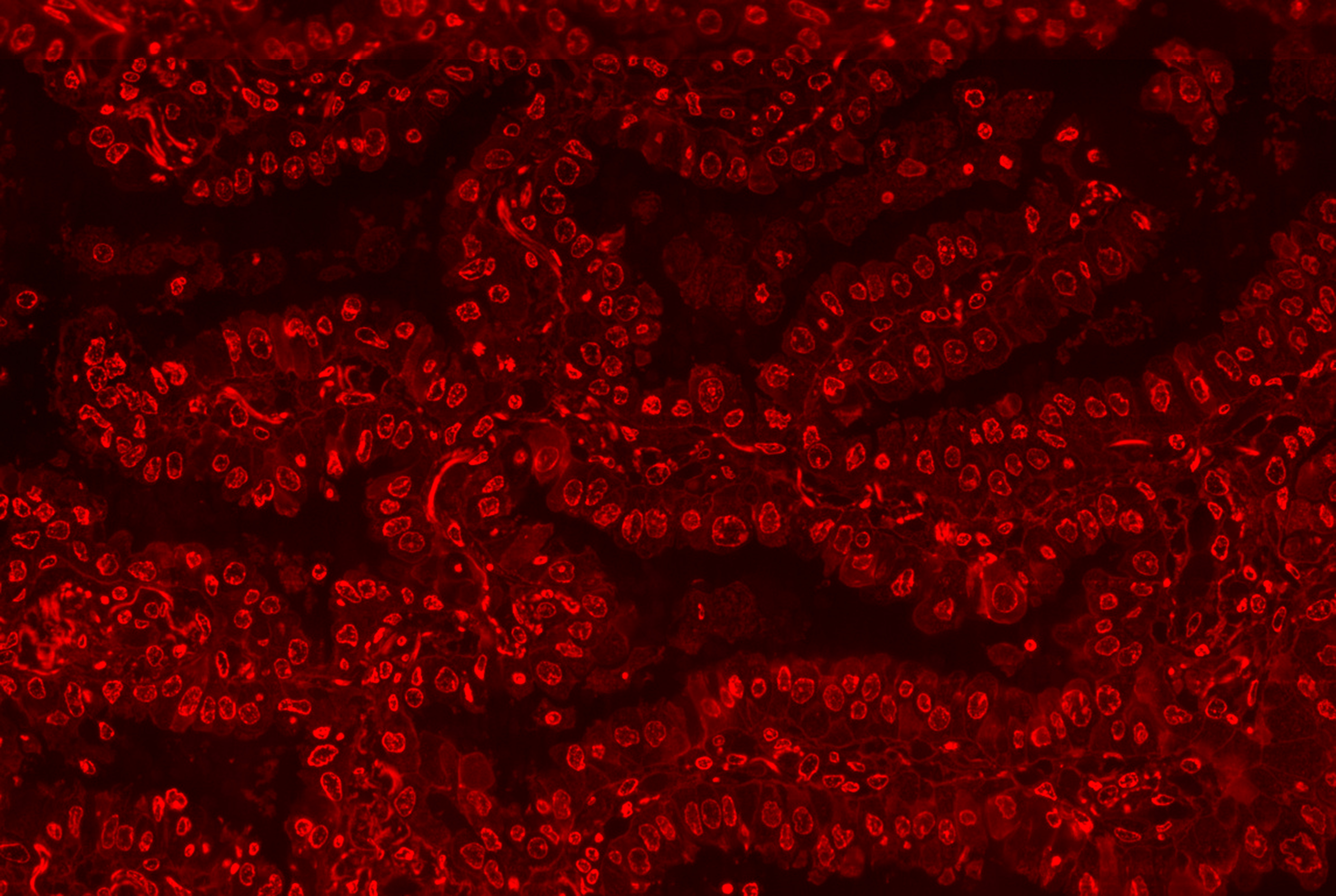

Supplement: Supplementary file 2 — Additional file 2. Imaging of cytoarchitecture channel of human lung adenocarcinoma tissue. [file 12938_2023_1072_MOESM2_ESM.jpg]
